# Transforming harmful representations, investigating violence and empowering care practises in masculinities

**DOI:** 10.3389/fsoc.2024.1416303

**Published:** 2024-08-27

**Authors:** Tatiana Moura

**Affiliations:** Centre for Social Studies, University of Coimbra, Coimbra, Portugal

**Keywords:** feminist theory/theories, non-violence, caring masculinities, empathy, intersectionality

## Abstract

Stemming from a critical perspective of feminist studies on masculinities, this article proposes to explore how dominant and hegemonic masculinities are being reimagined, renegotiated and reconstructed, by understanding new patterns of violence amongst boys, on the one hand; and how care, as the opposite of violence, and a concept in construction from a feminist perspective, can be adopted, put in practise and understood. From a feminist standpoint masculinities studies need to go through a renewed problematisation of the social constructions of masculinities in a space–time context that intersects the economic crisis, health crisis and, on the other hand, implementation of legislation and initiatives for gender equality and citizenship, and is marked by social challenges that are related to the increase of gender inequality, violence indicators and extremism but also the emergence of caring masculinities that need closer attention. This feminist approach to masculinities allows us to critically challenge hegemonic models of boyhood/manhood, specifically by developing a FEMINIST THEORY OF CARING MASCULINITIES. Through a critical understanding of the new patterns and ways in which patriarchal masculinities are perpetuated in society, and placing CARE as the centrepiece and pro-active practise that opposes violence sustained models of masculinities, feminist masculinities studies can challenge dominant forms of masculinity by taking action in diverse social contexts and emphasising empathy, emotional expression, and cooperative relationships.

## 1 Introduction

Recent political and theoretical approaches have raised alarms about the importance of combating harmful norms, attitudes and beliefs, including strategies that promote empathic attitudes and men’s involvement in caregiving practises. The preliminary findings of this research show that in semi-peripheral countries of the EU, like Portugal, elements that are usually associated with the current causes of violent masculinities are harboured, such as the disempowerment of the male provider that occurred sharply during the euro crisis and the high socio-economic impact of COVID-19.

The article is based on theory and research carried out under the scope of international projects (X-MEN^1^ and EMiNC – Engaging Men in Nurturing Care).^2^ Old and new patterns in violent attitudes, methods and behaviours were found among boys, on the one side, and care reconceptualizations and practises were identified that can contribute to revealing how care masculinities are built in everyday life, and serve as evidence to produce theoretical knowledge on feminist studies on masculinities.

## 2 Why do we need a feminist theory on caring masculinities? An introduction

Feminist theorists, as well as gender studies, have criticised dominant masculinity for reinforcing gender inequality and perpetuating violence against women (Connell, 1987, 1995; Messner, 1990; Higate, 2003; Hoffman, 2011; Baaz and Stern, 2013). It is argued that traditional forms of masculinity rely on the subordination and oppression of women and other marginalised groups, including men who do not conform to these hegemonic norms (e.g., Brod and Kaufman, 1994; Barker, 2005), and that these harmful norms and power dynamics contribute to gender inequality and violence. One of the central concepts in the study of masculinities is hegemonic masculinity (Connell, 1995), often linked to dominance of economic and political power.

While feminist masculinities theory proposes promoting non-violent masculinities as the opposite of violent ones in the context of gendered expectations and norms around masculinity (Flood, 2015; Jewkes, Flood, and Lang, 2015), some work (Borde et al., 2020; Moura and Alonso, 2021; Moura et al., 2021) proposes that care should be the focus and that masculinity and care are not mutually exclusive.

Feminist theory and practises on caring masculinities are, thus, an alternative to traditional forms of masculinity that seek to challenge and transform rigid gender norms, power imbalances and systemic oppression that impact the lives of both women and men.

Understanding what leads men and boys to prioritise caregiving pathways and equitable versions of masculinities is key to reshaping the feminist agenda on masculinities.

The main objective of this article is, on the one hand, to theorise about the social construction of masculinities in a space–time context characterised by a gradual crisis period and in contexts of privation of liberty (such as youth detention centres, under project X-MEN)^3^ and the potential of promoting care as a centrepiece in the period of these young people’s lives.

This article will explore three hypotheses:

The first is that crises (resulting from pandemics, war and the neoliberal economy) exacerbate extremely violent behaviours, and that these (more visible) behaviours affect mainly women and girls, but also men and boys, influencing expectations about masculinity. We need to understand the changing nature and adaptability of violence by analysing its new forms, patterns and tools.

The second is that crises and their implications can simultaneously transform care practises, fostering new changes in how gender identities are understood and performed, and can thus contribute to more equitable (more invisible) masculinities at the individual and societal levels.

Thirdly, non-violence and care are both important, but they are fundamentally different concepts, and care contributes more substantially to reducing violent masculinities.

## 3 Discussion

### 3.1 Care and non-violence as different practises

The relationship between care and the reduction of violent masculinities is a complex subject that has already been studied (Taylor et al., 2016; van der Gaag et al., 2023, among others). It is important to recognise that non-violence and care are indeed distinct concepts, and their contributions to reducing violent masculinities can vary depending on the context and approach taken. There is a need to recognise ways in which the dimension of care, as opposed to violence (rather than non-violence), can contribute to this reduction. First among these is reframing masculinity. Care challenges traditional and extremist notions of masculinity that often glorify aggression and dominance. This is particularly important at a time of public and virtual demonstrations of hatred towards women and the feminist movement. Recent literature on incels (involuntary celibates) underscores a growing concern about the socio-psychological dynamics that lead individuals to this identity and the potential for extremist behaviour. Researchers have highlighted the need for a nuanced and empathetic approach to addressing the underlying issues faced by incels, rather than defaulting to punitive or conservative responses.

Research by Hoffman et al. (2020) explores the cultural and psychological factors contributing to the incel phenomenon. They argue that societal pressures and unrealistic standards of masculinity and attractiveness play significant roles in the development of incel identities. This is compounded by the increasing influence of online communities where negative and extremist ideologies can proliferate without much opposition. A study by Scaptura and Boyle (2020) delves into the psychological aspects, finding that many incels exhibit traits of narcissism, entitlement, and a profound sense of victimhood. These characteristics can drive individuals towards extremist ideologies as a form of coping mechanism or as a way to find community and validation.

In this sense, given the complex interplay of psychological, social, and cultural factors, a care-based approach is increasingly advocated by scholars as an alternative to more punitive or conservative measures. A care-based approach not only helps individuals but also works towards mitigating the potential for extremist ideologies to take root.

By promoting care as a positive and essential attribute of masculinity, it encourages men to redefine their identities in ways that are not centred on violence or aggression. This reframing can be a powerful strategy in reducing violent behaviours.

To move forward with the proposal to promote care in order to redefine masculinities, we need to define care itself. The notion of care can be problematic in social research, particularly within the context of examining masculinities, for several reasons: (a) because of historical gendered associations – care has traditionally been associated with femininity, while dominance and aggression have been associated with masculinity. This binary understanding of gender roles has contributed to the marginalisation and devaluation of care within discourses of masculinity; (b) normalisation of dominant masculinities – promoting care as an alternative to violence challenges entrenched notions of dominant and hegemonic masculinities, characteristics such as emotional detachment. This can be met with resistance from individuals and institutions invested in maintaining existing power structures; (c) socialisation processes – boys and men are often socialised into patterns of behaviour that prioritise toughness and assertiveness over caregiving and emotional expression. In fact, reimagining masculinity to include care requires challenging deeply ingrained social norms and expectations. Intersectionality can also be a challenge: sexual orientation, expression, identity can complicate the notion of care; and different social groups may experience and express care differently, and approaches to caregiving may be influenced by broader structural inequalities.

Despite these challenges, the concept of “care” has been extensively analysed from various academic perspectives, including feminist theory and masculinities studies. Each offers unique insights into the importance and implications of care in social and personal contexts. Feminist theorists have long emphasised the importance of care as a fundamental aspect of human life and social organisation. They argue that care is often undervalued in patriarchal societies that prioritise economic productivity over relational and emotional well-being. Joan Tronto defines care as a “species activity that includes everything we do to maintain, continue, and repair our ‘world’ so that we can live in it as well as possible” (Tronto, 1993). This broad definition encompasses not only direct care activities, such as caregiving for children or the elderly, but also the maintenance of social and environmental conditions. Feminist scholars like Carol Gilligan (1982) have critiqued traditional moral theories for neglecting the ethics of care. Gilligan’s work highlights how care, characterised by empathy and relational thinking, contrasts with justice-oriented approaches that emphasise rules and impartiality. Furthermore, Nancy Fraser (2016) argues for the recognition of care work as essential labour that should be supported and fairly compensated within the economic system. Fraser’s analysis situates care within the framework of social justice, advocating for a reorganisation of societal priorities to better value care work. At the national level, we need to acknowledge the contributes of Pintasilgo (1987, 1995, 1999), a notable Portuguese feminist and politician. She contributed significantly to the discourse on care and caring theory by emphasising the ethical and social dimensions of care, advocating for a society that prioritises human dignity and relational well-being.

On the other side, masculinities studies examine how cultural norms around masculinity influence men’s behaviours and attitudes, including their approaches to care. R.W. Connell’s concept of hegemonic masculinity refers to the dominant form of masculinity that values traits such as toughness, emotional restraint, and independence (Connell, 2005). This often discourages men from engaging in caregiving roles, which are perceived as feminine. The pressure to conform to hegemonic masculinity can lead to an undervaluing of care both personally and societally. In fact, some scholars advocate for redefining masculinity to include caregiving as a valued and normalised behaviour. For instance, Jeff Hearn (2002) discusses the importance of promoting “caring masculinities” which encompass qualities like empathy, emotional expressiveness, and nurturing.

Altogether, and in sum, both feminist and masculinities studies underscore the necessity of revaluing care within society.

Encouraging care fosters emotional intelligence and empathy, qualities that can contribute to men better understanding and managing their emotions. When men are more in touch with their emotions and can express themselves in non-violent ways, they are less likely to resort to violent behaviours when faced with conflict or stress.

Care is also a foundation for building healthy, respectful relationships. Men who prioritise care are more likely to engage in positive, non-violent interactions with others. This can lead to healthier, more equitable relationships, reducing the incidence of violence within families, communities, and society at large.

Also, emphasising care can help prevent gender-based violence (GBV). By recognising the dignity and worth of all individuals, regardless of their gender, men can become allies in the fight against violence targeted at women and other groups. Men who embody care can serve as positive role models for others, especially younger generations. When boys and young men see caring and non-violent behaviour displayed by their male role models, they are more likely to adopt similar behaviours and reject violent masculinities.

A culture of care can contribute to broader social and cultural changes that reject violence as an acceptable means of asserting power or control. This can lead to the development of policies and programmes aimed at reducing violent behaviours and promoting care as a societal value.

However, it is essential to recognise that care alone may not be sufficient to address all aspects of violent masculinities. Combining care with a commitment to non-violence can provide a more comprehensive approach. Non-violence actively opposes and rejects violent behaviours, while care focuses on the positive attributes and behaviours that can replace violence, and thus have a pro-active role.

In summary, care can play a significant role in reducing violent masculinities by challenging traditional norms, promoting emotional intelligence, fostering healthy relationships, preventing GBV, providing positive role models, and contributing to broader social change. However, a comprehensive approach may involve combining care with a commitment to non-violence to address the issue from multiple angles.

## 4 New patterns of violence and patterns of care: engaging men in feminist theory of care

### 4.1 New patterns of (gender-based) violence amongst young people in Portugal

As in many other countries, young people in Portugal express new and changing violent behaviours that can be related to several factors: they may have been increasingly exposed to online violence and harassment, including cyberbullying; they may be victims of bullying in schools; they may consume anti-feminist online contents that contribute to the way they construct their identities (and masculinities in particular). In our X-MEN research, we explored some possible patterns and considerations related to GBV among young people in Portugal. Digital GBV, for example, has increased in recent years. This includes online harassment, cyberbullying, revenge porn, and other forms of online abuse targeting individuals based on their gender.

In fact, the digital environment presents unique challenges in addressing and preventing such forms of violence. The National Study on Dating Violence 2023,^4^ carried out by Union of Alternative and Responsive Women (UMAR, 2023), as part of the ART-THEMIS + project, has been carried out annually since 2017 and analyses the legitimization of dating violence by young people and the indicators of victimisation in dating relationships. It involves young people from the 7th to the 12th grade, from regular or vocational education and from various schools across the country. Of all the young people taking part in the study, 67.5% did not perceive at least one of the following behaviours as dating violence: control, psychological violence, sexual violence, stalking, violence via social networks and physical violence.

The results of this study point to the importance of primary prevention of GBV in the school context, which should be developed in a holistic, systematic and ongoing way, in order to raise awareness among children and young people of the need to deconstruct violence and develop healthy interpersonal dating and intimate relationships.

In some urban areas, there have been concerns about youth involvement in gang-related violence (data from X-MEN), which can include activities like drug trafficking, vandalism, and turf wars. Economic and social factors may contribute to the appeal of these activities for some young individuals.

Concerning the private sphere, young people may also be exposed to violence within their families or may be perpetrators themselves. This can be due to various factors, including family behaviours, intergenerational violence, or economic stress. These factors affect mostly the ways boys build their masculinities and cannot be only associated with young people living in the margins of society. In fact, there are already very interesting analyses by Monteiro (2023) about the ways peripherical masculinities are built, in order to achieve status, visibility and respect.^5^

Socio-economic factors, such as social inequality, poverty, unemployment, and limited access to education and opportunities, can contribute to violence among youth. But these factors may vary by region and community.

The Portuguese government and various NGOs have been working on preventive measures to address youth violence. This includes educational and gender transformative programmes, community initiatives, and support services for what they consider at-risk youth. The project X-MEN – Masculinities, Empathy and Non-Violence, carried out by some authors of this special issue, is one example. The project’s main objective is to promote non-violent and equitable masculinities and the development of strategies that break the cycles of violence, including gender-based violence (GBV). The project was implemented in three European countries – Portugal, Spain and Croatia. During adolescence and early adulthood, gendered behaviours and attitudes are often influenced (and might be exacerbated) by the institutional contexts where the risk of being exposed to symbolic and actual violence is considerably higher, such as juvenile detention centres and refugee camps. Most of these contexts are hierarchical institutions ‘sealed off’ from the outside world, and present challenges to promoting empathy and equitable gender relations.^6^

Mental health issues among young people can also be a contributing factor to violence, especially after the lockdowns imposed during COVID-19. A study by the Organisation for Economic Co-operation and Development (OECD) (2021)^7^ affirms that mental health problems have increased in Europe as a result of the pandemic and that this is a particularly serious development among young people, who are now more affected by depression than the entire adult population. In its comparative report on the health situation in Europe, the OECD explains that ‘while in 2019 the percentage of young people aged between 15 and 24 with symptoms of depression was 6%, compared to 7% of adults,’ with COVID-19 the numbers have increased in all countries for which data is available. According to this organisation, the proportion of young people with symptoms of depression is now at least 50% higher than the general population and, in some countries, twice as high.^8^

According to the statistics available (from 2020 and 2021), in Norway, young people with depression increased to 42.5 per cent, compared to 17.1 per cent for the adult population; in Austria, the percentage reached 41.3 per cent (23.7 per cent in adults) and in Sweden it reached 38.5 per cent (17.1 per cent in adults). In Spain, 35 per cent of young people showed signs of depression, compared to 22.5 per cent of adults; in the United Kingdom, the percentage was 30 per cent in young people and 15 per cent in adults; in Italy, young people with depression were 24.2 per cent and adults 14.4 per cent, while in France 20.1 per cent of young people and 16.5 per cent of adults had symptoms of depression. It is assumed that confinement and restrictions on movement have disproportionately harmed young people psychologically.

Addressing mental health needs and providing access to appropriate services is thus crucial. Bullying in schools and among peer groups can take on gendered forms, such as sexual harassment, homophobic or transphobic bullying, and gender-based taunting. However, GBV among young people is not experienced uniformly. It is important to consider how factors like race, sexual orientation, gender identity, and socio-economic status intersect with gender to shape young people’s experiences of violence. It is undeniable, however, that GBV can have significant psychological and emotional effects on young survivors. Addressing the mental health and trauma-related aspects of violence is crucial.

## 5 Engaging men in feminist theory and (new) practises of care

Engaging men in a feminist theory and practises of care requires a nuanced approach that acknowledges new patterns of both violence and care. There are key considerations for incorporating men into feminist discussions and practises of care, like recognising intersectionality, for example, which acknowledges the interconnectedness of various forms of oppression and privilege. When engaging men in discussions of care, it is important to recognise that men’s experiences and roles in care and violence may be influenced by factors such as race, class, sexual orientation, and disability. When working on masculinities and the promotion of caring masculinities, we need to consider that these intersections help create a more inclusive and comprehensive understanding.

With these intersections in mind, we can challenge traditional notions of masculinity, which often emphasise dominance, emotional suppression, and the use of power and control. Engaging men in feminist care involves challenging these traditional constructs and encouraging men to embrace a more caring and empathetic version of masculinity. This can be achieved through public policies and programmes focusing on gender-inclusive early education, awareness campaigns and positive role modelling, among other initiatives.

Also, in order to engage boys and men in nurturing care entails understanding the above-mentioned new forms of violence. Contemporary forms of violence may manifest differently from traditional forms that need to be addressed. Engaging men in feminist discussions of care involves understanding and addressing these new patterns of violence, like online violence or cyberbullying, including how they intersect with gender dynamics.

Care is inherently tied to empathy and healthy and respectful relationships. Engaging men in feminist care should emphasise the importance of consent, communication, and mutual respect in all relationships, whether intimate, familial, or social.

From our research and that of our partners (van der Gaag et al., 2023; Moura, 2024) and their projects, men’s involvement in caregiving roles, such as parenting and eldercare, should be encouraged and supported. Policies and workplace practises that promote work-life balance and shared caregiving responsibilities can help break down traditional gender roles and foster more caring and equitable societies.

Also, collaboration with male allies and engaging men in supporting and having an active role in care practises can be highly effective. Men who actively support gender equality and care can work alongside women and feminists to challenge harmful norms, advocate for policy changes, and promote cultural shifts towards care and non-violence.

Incorporating men into feminist theories and practises of care is essential for achieving gender equality and reducing violence in society. It involves breaking down traditional gender roles and fostering a culture where care, empathy, and non-violence are valued by people of all genders. Ultimately, engaging men in these discussions is about promoting a more just and equitable world where everyone can live free from violence and discrimination.

My proposed feminist approach seeks to address these issues by reconceptualizing masculinities and placing care at the forefront. By advocating for a feminist theory of caring masculinities, my objective is to contribute to the disruption of dominant models of masculinity and promote more equitable and compassionate forms of gender identity. This involves not only understanding and analysing the social constructions of masculinities but also actively working to challenge and transform them through legislative and initiative-based interventions.

## 6 Conclusion

### 6.1 Spaces of resistance: engaging men and fathers from early childhood

Data from the World Health Organization (WHO) affirms that violence against children includes all forms of violence against people under 18 years old, whether perpetrated by parents or other caregivers, peers, romantic partners, or strangers. Globally, it is estimated that up to 1 billion children aged 2–17 years have experienced physical, sexual, or emotional violence or neglect in the past year.^9^ Experiencing violence in childhood impacts lifelong health and well-being. But evidence from around the world shows that violence against children can be prevented.

### 6.2 Types of violence against children

Most violence against children involves at least one of six main types of interpersonal violence that tend to occur at different stages in a child’s development, and when directed against girls or boys because of their biological sex or gender identity, any of these types of violence can also constitute GBV.

Violence against children has lifelong impacts on the health and well-being of children, families, communities, and nations. A lack of emotional bonding between children and parents or caregivers can lead to long-term impacts. But the good news is that violence, and in particular violence against children, can be prevented. Preventing and responding to violence against children requires efforts to systematically address risk and protective factors at all four interrelated levels of risk (individual, relationship, community, society).

Engaging men and fathers from early childhood is crucial for several reasons, as it can have a significant positive impact on children, families, and society as a whole. Engaging men in caregiving roles challenges traditional gender norms and promotes gender equality. When men actively participate in childcare from the earliest stages, it sends a powerful message that caregiving is not exclusively a woman’s responsibility. This helps break down gender stereotypes and expectations.

It also enhances child development (IMAGES,^10^ SOWF).^11^ Fathers’ involvement in early childhood contributes to children’s cognitive, emotional, and social development. Studies have shown that children with involved fathers tend to perform better in school, have higher self-esteem, and exhibit better emotional regulation. Building strong bonds between fathers and their children from an early age lays the foundation for healthy parent–child relationships. These positive attachments provide emotional security and support children’s overall well-being.

Also, one of the most important consequences is the balance between work-life responsibilities. Encouraging fathers to be actively engaged in caregiving can lead to more equitable sharing of household and childcare responsibilities. This balance allows mothers to pursue their careers and interests while fathers take on a more active role in raising children. When fathers are involved in their children’s lives and model respectful behaviour in their relationships, it reduces the likelihood of violence within families. Children raised in non-violent households are less likely to perpetrate or tolerate violence in their own relationships in the future (IMAGES).

As already mentioned, active role models are crucial for boys and children. Active fathers serve as positive role models for their children, demonstrating the values of empathy, communication, and cooperation. This modelling of healthy behaviours can have a long-lasting impact on how children perceive and engage in relationships.

The positive effects of engaging men and fathers from early childhood ripple out into society, leading to more equitable communities. When children grow up with involved fathers, they are more likely to become responsible and empathetic adults, contributing positively to society.

In conclusion, engaging men and fathers from early childhood is not just about changing family dynamics but also about promoting gender equality, enhancing child development, reducing violence, and building stronger, more inclusive communities. It is an essential step towards creating a more equitable and nurturing society for everyone. It is central to new definitions, theories and practises of care.

## 7 Emerging trends and new patterns of care among young people

There are emerging trends and discussions regarding new patterns of care among young people, including boys. These patterns were reflective of evolving societal norms, generational shifts, and changing attitudes towards caregiving roles. However, we need to keep in mind that these trends continue to evolve or change over time. Some potential new patterns of care among young people, including boys, can be the previously mentioned involvement in parenting. Some boys and young men have been increasingly involved in parenting and caregiving activities. This includes activities like diaper changing, feeding, and spending quality time with their children. This shift reflects a growing recognition of the importance of shared parenting responsibilities.

Also, young boys are increasingly embracing emotional expression, although still not sufficiently. Boys are being encouraged to express their emotions and develop emotional intelligence and this can lead to a more caring and empathetic approach to relationships and caregiving. The breaking down of traditional expectations that boys should be ‘strong’ and unemotional is a positive development.

Furthermore, from our projects and research (X-MEN) we can affirm that young people, including boys, often provide emotional support and care to their friends. They may be more open to discussing personal issues and offering help when needed. This shift can promote mental health and well-being among peers.

Concerning activism and social justice, we can see many young people all over the world, including boys, engaging in activism and advocacy for social justice causes. This can involve caring for and supporting marginalised communities, promoting inclusivity, and advocating for positive social change. One of the strategies used by young people are online communities and social media platforms, which have provided them with spaces to seek and offer support, advice, and care. Young people may participate in online support groups or use social media to connect with others facing similar challenges.

Our KINDER project^12^ and other international programmes, such as the Global Boyhood Initiative,^13^ showed us the increasing importance of schools and educational institutions, which increasingly emphasise the importance of care, empathy, and healthy relationships. Educational programmes may address topics like consent, respect, and emotional well-being.

Some young boys and men are also navigating caregiving responsibilities alongside educational pursuits and careers. Balancing these roles can require innovative strategies and support from employers and educational institutions.

It is important to note that these new patterns of care among young people are not universal, and they can vary significantly depending on cultural, socio-economic, and individual factors. Additionally, these trends may continue to evolve as societal norms and expectations change over time.

## 8 Main challenges for masculinities in boys in juvenile detention centres: the X-MEN project in Portugal

Youths in juvenile detention centres face a range of challenges related to masculinities, which can impact their experiences, behaviours, and reintegration. They can adhere to violent and dominant masculinities – many boys in detention centres may have been influenced by violent masculinity norms, which can encourage behaviours such as aggression, dominance, and emotional suppression. Detention centres need to address these harmful norms to promote healthier expressions of masculinity.

Some youths in detention may have a (family) history of violent behaviours, often associated with their attempts to conform to traditional notions of masculinity. Detention centres must work to address these violent tendencies and provide alternative ways to resolve conflicts.

We need also to consider the context. The environment in detention centres can be highly influenced by peer dynamics. Youth may feel pressure to conform to certain masculine stereotypes to gain respect or avoid victimisation. Creating a supportive, empathetic and non-violent atmosphere is essential.

Many youths in detention have experienced trauma, which can affect their mental health and exacerbate issues related to masculinity. Addressing trauma and providing mental health support is crucial for adhering to healthier masculinities. Boys in detention centres may struggle with expressing their emotions due to societal expectations of stoicism. Encouraging emotional expression and teaching healthy coping mechanisms is vital.

Self-esteem and identity were central subjects crosscutting the entire X-MEN programme. Youths in detention may grapple with self-esteem and identity issues, particularly if they perceive their actions as failing to align with traditional notions of masculinity and/or see no valuable future in their lives.

Detention centres offer educational and vocational training, but they should not reinforce stereotypes about what careers are considered ‘masculine’ and ensure that boys (and youth) can pursue their school life after leaving the detention centres. This can help youths envision alternative paths for themselves. Preparing young people for reintegration into society includes addressing how they perceive and perform masculinity. They may face challenges in readjusting to societal expectations and peer pressures outside of detention.

In the case of youth in juvenile detention centres, providing positive male role models and mentors who can demonstrate healthy expressions of masculinity is absolutely crucial for youths to learn alternative behaviours and values.

Also from our research, family can play a significant role in shaping a young person’s understanding of masculinity and life. Detention centres should work with families to address issues related to masculinity and provide support and education.

By challenging violent masculinity norms, providing emotional support, and fostering positive male identities, detention centres can contribute to the rehabilitation and successful reintegration of young people into society.

## 9 Masculinities, empathy and non-violence: breaking cycles of violence and promoting equitable and caring relationships

### 9.1 Masculinities and the intergenerational transmission of violent behaviour

Exposure of children and young girls and boys to violence (e.g., domestic violence, corporal punishment, community violence, school violence, war and post-war violence) can lead to the normalisation of violent behaviour, including GBV and a range of mental health problems, as well as influence the use of violence as adults (Taylor et al., 2016; Till-Tentschert, 2017).

Exposure to violence can be defined as direct physical aggression, threats of physical and psychological harm, or being a witness to it and can have implications for trajectories of anxiety and depressive symptoms with a described impact on young people’s health when they reach adulthood (Felitti et al., 1998; Heinze et al., 2017). A study by the Johns Hopkins Bloomberg School of Public Health (Blum et al., 2019) showed that 46% of the young adolescents in the study reported having experienced violence. The report also pointed out that boys are more likely to adopt more violent behaviour as adults due to rigid gender norms, while girls tend to have higher levels of depression.

According to the European Commission Study on the Role of Men in Gender Equality (2013), the perpetration of violence by men or their victimisation depends on age (Belghiti-Mahut et al., 2012), and violent behaviour and the normalisation of violence can, in some cases, be transmitted generationally (Belghiti-Mahut et al., 2012; Till-Tentschert, 2017). The link between the exposure of young people (male and female) to violence and its intergenerational transmission is now clearly established. Both witnesses and victims of violence have similar associations with aggression and the perpetration of violence in its many forms. For example, the International Men and Gender Equality Survey on Urban Violence – IMAGES, which took place in Rio de Janeiro and Maputo (Taylor et al., 2016), found that exposure to urban violence before the age of 18 was strongly associated with perpetrating violence as an adult. Exposure to violence outside the home was also shown to be highly related to the use of violence in the home (violence is also related to attitudes towards gender equality, with both men and women questioned in the survey showing less gender-equitable attitudes where exposure to violence was greater) (Moura et al., 2024).

Similarly, Belghiti-Mahut et al. (2012) have also shown that violence is often influenced by the institutional contexts of these boys and men. For example, although violence occurs in (almost) all spheres of society during adolescence (12–18 years old), it can be exacerbated in specific institutions where the risk of exposure to violence and rigid gender norms is considerably higher than elsewhere, such as in military contexts, prisons, educational centres, etc. These are mostly hierarchical institutions, quite ‘isolated’ from the outside world, thus presenting a challenge to current strategies for promoting gender equality.

More recently, the goal of breaking cycles of violence has been linked to efforts to promote positive, non-violent and caring relationships, thus targeting rigid gender norms, including masculinity. Various efforts have been made to involve men and masculinities within the framework of the Agenda for Gender Equality in Europe (Gender Equality Strategy 2020–2025; European Commission’s Strategic Engagement for Gender Equality 2016–2019, Stockholm Programme 2010–2014, EU Regulation No. 6060/2013, Directive 2012/29/EU. 2011/99/EU, 2011/36/EU, Istanbul Convention [2016], Directive 2011/99/EU on the European Protection Order [EPO], etc.).

Given this background, X-MEN emphasised the need to target at-risk or socially excluded young boys and men who have been victims or witnesses of violence and/or perpetrators of violence, within the framework of actions and interventions aimed at combating GBV and promoting gender equality. However, due to the complexity of this framework, the gender transformative programmes used were both gender and age sensitive, co-developed in a participatory way with the voices of young people, and sustainable (for example, by integrating a gender perspective into these institutions).

## 10 Why X-MEN?

### 10.1 Fundamentals of the X-MEN project

X-MEN chooses to focus on masculinities because, as previously mentioned, men and boys have an essential role to play in the prevention of violence and in the intergenerational transmission of violent/non-violent behaviour (CARE Int., 2012). In addition, we chose a young audience at risk (vulnerable children aged 12 to 16), since early intervention is key to breaking cycles of violence and preventing recidivism (in situations where these minors are the promoters of violence). In addition, X-MEN has an interest in understanding how COVID-19 has had an impact on gender expressions, gender relations, gender behaviours, etc. among these groups.

X-MEN’s focus on young men and masculinities is supported by the recent Bellagio Working Group Report on Gender Equality (2019), which affirms the importance of intervening from an early age to include boys in strategies to promote gender equality and non-violence. The active involvement of young boys is crucial, given that it is at this age that gender norms, attitudes and beliefs tend to become rigid. In X-MEN, it is thus considered that focussing on young people in different contexts who have experienced complex forms of violence and from diverse backgrounds is essential to address the complexity of experiences and manifestations of gender equality and violence, targeting the link between exposure to violence and its perpetration, and the inverse relationship between violence and attitudes to gender equality (Alonso, 2015).

As far as Portugal is concerned, the project was designed around a group of young people considered to be at risk of social exclusion: ‘young people in conflict with the law’ in the tutelary education system. However, a sustainable strategy cannot be dependent on a ‘project-based approach,’ and the institutional establishment of practises and strategies to promote gender equality is vital for a future without GBV. In this sense, the X-MEN gender transformative approach will also target active professionals and experts (social workers, psychologists, monitors, carers, teachers, etc.) who work with our main youth target groups, in order to provide them with internationally evaluated tools to deal with adverse childhood experiences and promote positive, non-violent relationships targeting rigid gender norms.

In short, the main objective of X-MEN was to develop a set of synchronised, gender-reflective tools that address the construction of non-violent and empathetic identities of young boys and girls (12–18 years old).

## 11 How?

### 11.1 Concept

‘X-MEN – Masculinities, Empathy and Non-Violence’ is inspired by the comic books of fictional superheroes. In these comic books, X-MEN are a subspecies of humans who fight for peace and equality between ‘normal’ humans and mutants (difference in the norm). This conflict is often compared to real-world conflicts experienced by minority groups. In addition, X-MEN have always represented diversity, difference and misfits whose powers reach them in their teenage years, making them the perfect model and analogy for the young boys and girls targeted by this project.

In general, X-MEN co-develops methodologies and tools with the target groups of young people that respond to adverse childhood experiences, promote positive and non-violent relationships, target rigid gender norms, and build a cross-country advocacy strategy. X-MEN is built on the assumption that changing rigid gender norms, particularly masculinities, to promote gender inequality is increasingly recognised as an important intervention strategy and that gender-synchronised approaches will make it possible to deal with interpersonal and structural violence. X-MEN uses the analogy of mutants to foster gender norm-transforming interventions aimed at changing individual knowledge, attitudes and behaviours while seeking to break the intergenerational cycle of violence.

## Ethics statement

The studies involving humans were approved by Ethics Committee of the Centre for Social Studies, University of Coimbra. The studies were conducted in accordance with the local legislation and institutional requirements. Written informed consent for participation in this study was provided by the participants’ legal guardians/next of kin. Written informed consent was obtained from the individual(s) for the publication of any potentially identifiable images or data included in this article.

## Author contributions

TM: Writing – original draft.

## Footnotes

^1^X-MEN – Masculinities, Empathy, Non-violence was funded by the European Union’s Citizens, Equality, Rights and Values programme (2021–2027) under grant agreement 101049457.

^2^https://www.issa.nl/content/engaging-men-nurturing-care

^3^https://xmen.ces.uc.pt/

^4^https://www.cig.gov.pt/2023/02/apresentacao-do-estudo-nacional-sobre-violencia-no-namoro-2023/

^5^https://gerador.eu/a-afirmacao-da-masculinidade-em-contextos-perifericos/

^6^https://xmen.ces.uc.pt/

^7^https://www.oecd.org/coronavirus/policy-responses/supporting-young-people-s-mental-health-through-the-covid-19-crisis-84e143e5/#contact-d4e2442

^8^OECD Policy Responses to Coronavirus (COVID-19), Supporting young people’s mental health through the COVID-19 crisis, 12 May 2021.

^9^Global prevalence of past-year violence against children: a systematic review and minimum estimates. Hillis S, Mercy J, Amobi A, Kress H. Pediatrics 2016; 137(3): e20154079.

^10^https://www.equimundo.org/images-research/

^11^https://men-care.org/what-we-do/advocacy/state-of-the-worlds-fathers/

^12^https://kinder.ces.uc.pt/

^13^https://www.boyhoodinitiative.org/
